# Generation of alkyl and acyl radicals by visible-light photoredox catalysis: direct activation of C–O bonds in organic transformations

**DOI:** 10.3762/bjoc.20.119

**Published:** 2024-06-14

**Authors:** Mithu Roy, Bitan Sardar, Itu Mallick, Dipankar Srimani

**Affiliations:** 1 Department of Chemistry, Indian Institute of Technology-Guwahati, Kamrup, Assam 781039, India

**Keywords:** acyl radical, alkyl radical, sustainable catalysis, visible light

## Abstract

Alkyl and acyl radicals play a critical role in the advancement of chemical synthesis. The generation of acyl and alkyl radicals by activation of C**–**O bonds using visible-light photoredox catalysis offers a mild and environmentally benign approach to useful chemical transformations. Alcohols, carboxylic acids, anhydrides, xanthates, oxalates, *N*-phthalimides, and thiocarbonates are some examples of alkyl and acyl precursors that can produce reactive radicals by homolysis of the C**–**O bond. These radicals can then go through a variety of transformations that are beneficial for the construction of synthetic materials that are otherwise difficult to access. This study summarizes current developments in the use of organic photocatalysts, transition-metal photoredox catalysts, and metallaphotocatalysts to produce acyl and alkyl radicals driven by visible light.

## Introduction

The growing awareness of the necessity for sustainable developments has been heightened by the current energy crisis and the adverse impacts of industrialization. The development of green and energy-efficient methods in organic chemistry that use renewable sources of starting materials is considered highly sustainable [1–3]. Radical reactions have profound applications in organic synthesis [4–9]. In the context of sustainable catalysis, visible-light-mediated chemistry is becoming a prominent viable option for radical transformations in the synthesis of biologically useful compounds due to the energy efficiency and environmental friendliness [10–11]. Recently, the photocatalytic and electrochemical deoxygenation of acids and alcohols has attracted significant attention as the strategic cleavage of the C–O bond is quite challenging and opens up new possibilities for constructing useful compounds [12–14].

The use of photogenerated carbon-centered radicals, such as acyl and alkyl radicals, has shown great promise in the synthesis and functionalization of various organic molecules [15–16]. These carbon radicals can be generated in several ways. The first and most straightforward method is the homolytic cleavage of labile C–heteroatom bonds, especially alkyl halides [17]. Due to growing concerns about the harmful effects of toxic compounds (e.g., some halides) on the environment, the scientific community is now looking for alternative chemicals that can form these radicals. Moreover, discovering mild strategic approaches for the generation of unstabilized alkyl and acyl radicals and maintaining a high degree of selectivity with respect to the desired outcome are key obstacles to the growth of alkyl and acyl radical chemistry. With this in mind, the emergence of new chemical transformations involving radicals generated via C–O bond cleavage by visible light becomes significant.

**Figure 1:**
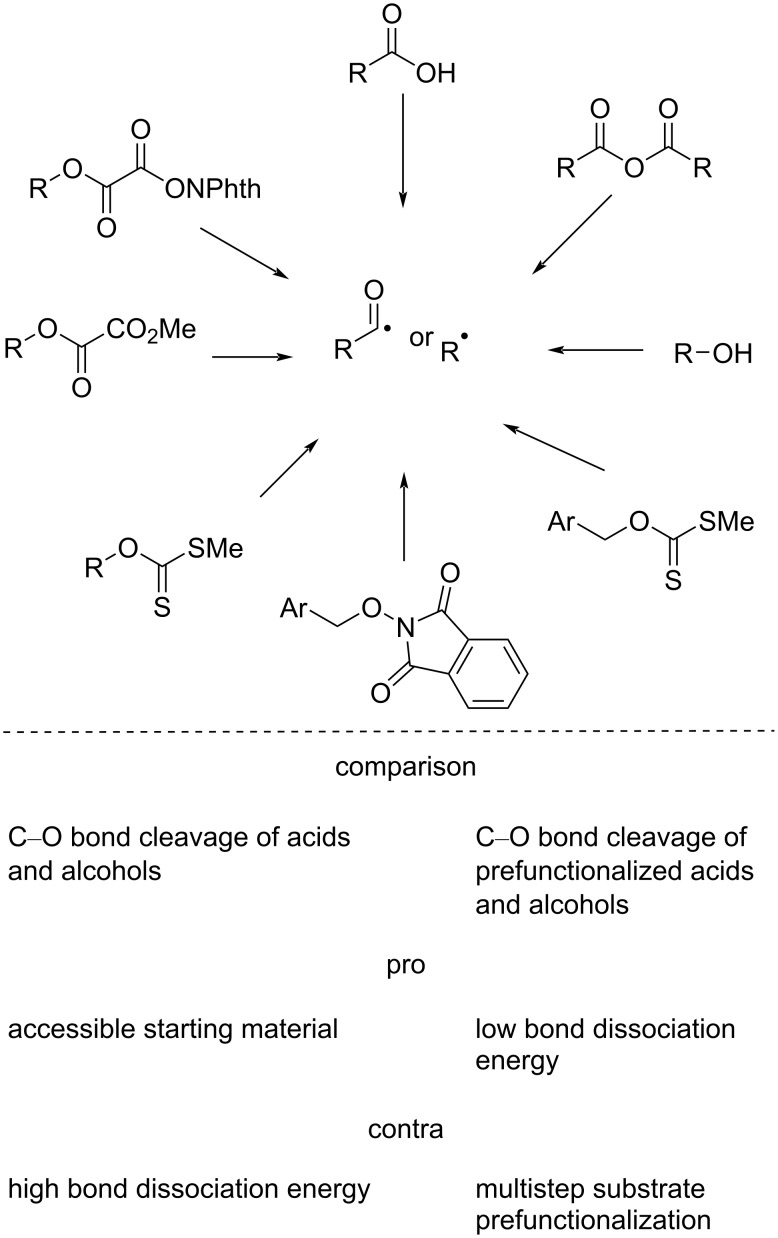
Generation of alkyl and acyl radicals via C–O bond breaking.

C(sp^3^)–O bonds are ubiquitous in nature and can be easily found in biochemical feedstocks, such as alcohols and acids. On the other hand, alcohols and acids are easily accessible. Although the starting materials are abundant, the C–O bond strength and the high redox potential impede the progress of photoredox-mediated catalysis in this domain [18]. Conventional procedures, such as Barton–McCombie deoxygenation [19] and use of thiocarbonyl-based activating groups [20], are quite popular. However, they have certain limitations, such as the use of toxic reagents, high temperature, and UV light [21], among others. Recent advancements include the photoinduced deoxygenation of acids and alcohols by means of anhydride, xanthate, carboxylate, oxalate, and *N*-alkoxyphthalimide functionalization (Figure 1) and utilization in visible-light-mediated chemical transformations. Despite the great importance of these strategies, the direct use of acids or alcohols is more fascinating as this approach circumvents the additional synthesis of special functionalized compounds. The strategy involves in situ activation by appropriate reagent, followed by photochemical C–O bond scission to directly access acyl or alkyl radicals from carboxylic acids or alcohols, thereby eliminating the need for an extra step. As a result, such methods are continuously gaining popularity.

This review delves into the current state of deoxygenation processes, focusing on techniques that use visible-light irradiation to harness the reactivity of alcohols and acids both upon derivatization and through direct use. By exploring recent advancements in deoxygenation reactions and the design of potential reactants, we aim to give an overview of the diverse strategies that highlight the unique reaction design and promote green chemistry principles. In this regard, in 2019, Banerjee et al. published a review article on the generation of acyl radicals in the presence of visible light [22]. Therein, they discussed the various ways in which visible light can generate acyl radicals from different organic molecules to facilitate important chemical reactions. Thus, we will focus on the reports detailing organic transformations that proceed via visible-light-induced deoxygenative generation of acyl radicals from carboxylic acids and acid anhydrides that have appeared since 2019.

## Review

### General mechanism of photoredox catalysis

**Figure 2:**
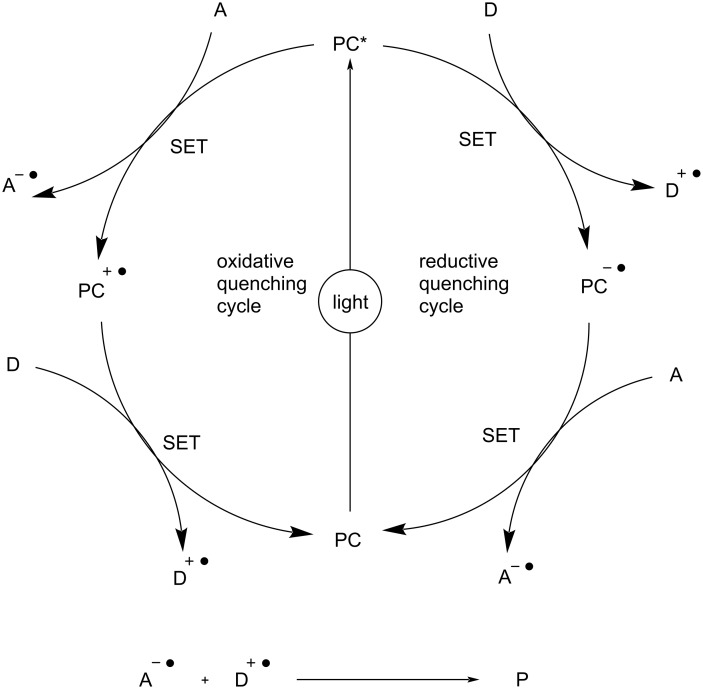
General photocatalytic mechanism.

In recent times, visible-light-mediated photoredox chemistry has evolved as a unique tool for various organic transformations. In contrast to traditional catalysis, the photochemical process uses an electron or energy transfer mechanism to form reactive intermediates. Typically, a photocatalyst is triggered to carry out energy transfer and electron transfer or proton-coupled electron transfer when it absorbs light of an appropriate wavelength (Figure 2). These processes generate highly reactive species, such as radical cations or anions, which can initiate the desired organic transformations. Different photocatalysts, such as transition metal complexes [23–24], organic dyes [25], and semiconductors [26], can be employed for visible-light-induced chemical processes. The choice of photocatalyst depends on the specific requirements of the catalytic process, including the type of reaction, the targeted absorption wavelength, and the overall efficacy. Researchers continue to explore and design photocatalysts to enhance the performance in various photocatalytic applications.

Visible-light-induced photoredox catalysis has been used in a variety of chemical reactions, including C–C, C–N, C–O, and C–halogen bond formation, as well as C–H functionalization [27]. Some notable examples include C–H arylation, various cross-coupling reactions, oxidative coupling, and photocatalytic radical reactions. The advantages of visible-light-induced photoredox catalysis are due to the ability to utilize visible light, a sustainable and abundant energy source, to initiate chemical reactions. This approach offers milder reaction conditions, which often result in improved selectivity and functional group compatibility. Additionally, it allows the activation of typically inert bonds and can enable the development of novel synthetic strategies. It has expanded the scope of available synthetic methods and contributed to the synthesis of complex molecules with high efficiency and selectivity. Ongoing research in this field continues to explore new catalysts, photosensitizers, and reaction mechanisms to further advance the field of visible-light-mediated organic transformations.

This review describes techniques for the generation of alkyl and acyl radicals by breaking the C–O bonds of various reactants with visible light and how to use them in organic transformations.

### Reactions involving acyl radicals obtained via C–O bond cleavage

#### Direct C–O bond activation of acids

**Scheme 1:**
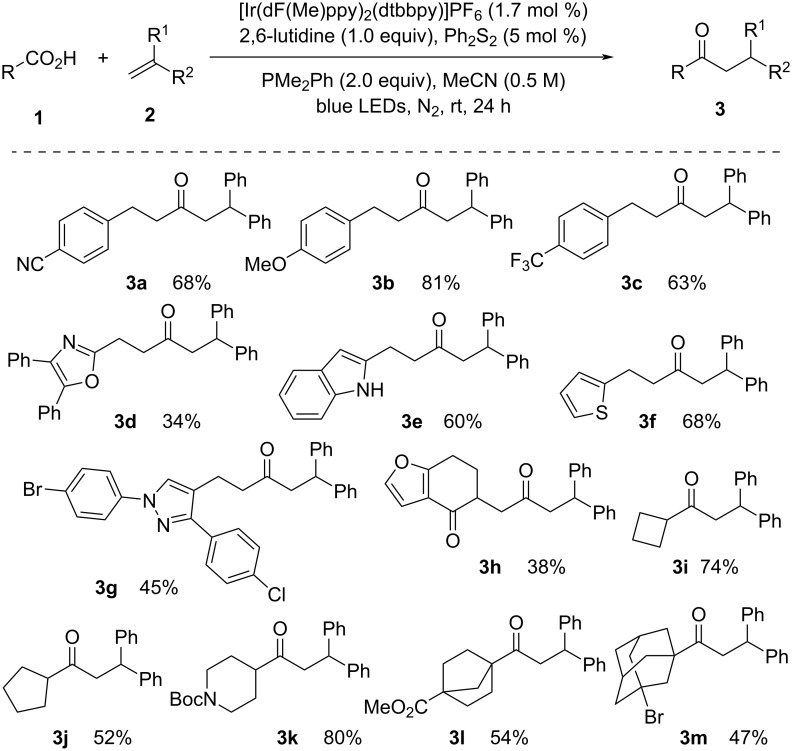
Photoredox-catalyzed hydroacylation of olefins with aliphatic carboxylic acids.

In visible-light photoredox catalysis, simple and affordable carboxylic acids and acid anhydrides can be utilized as good acyl radical sources for the deoxygenation approach. Acyl radicals can be generated through the use of photocatalysts, such as iridium complexes or organic dyes, and activating agents, including dimethyl dicarbonate (DMDC) and PPh_3_. Redox-active esters are created when activating agents and carboxylic acids react. These esters can then be reduced using a photoredox catalyst to produce the acyl radical. In 2019, Doyle [28] introduced the photoredox-catalyzed hydroacylation of styrene derivatives via deoxygenation of challenging aliphatic carboxylic acids (Scheme 1). The deoxygenation was promoted by phosphine reagents to form acyl radicals. The acyl radicals reacted with the C=C bond and formed the expected product. Appropriate selection of the phosphine reagent was the key to success in the process. Due to the lower oxidation potential, electron-rich PMe_2_Ph preferentially transferred a single electron to the excited state of the photocatalyst rather than the alkene, which was essential for obtaining the desired product in a satisfactory yield.

Various hydrocinnamic acids having electron-donating and electron-withdrawing substituents provided the targeted products **3a**–**c** in good yield. Heterocycles containing carboxylic acids also provided the products **3d**–**g** in moderate to good yield. Aliphatic carboxylic acids, under optimized or slightly modified conditions, cyclic secondary, and tertiary alkyl carboxylic acids were smoothly coupled with 1,1-diphenylethylene to give **3i**–**m**.

**Scheme 2:**
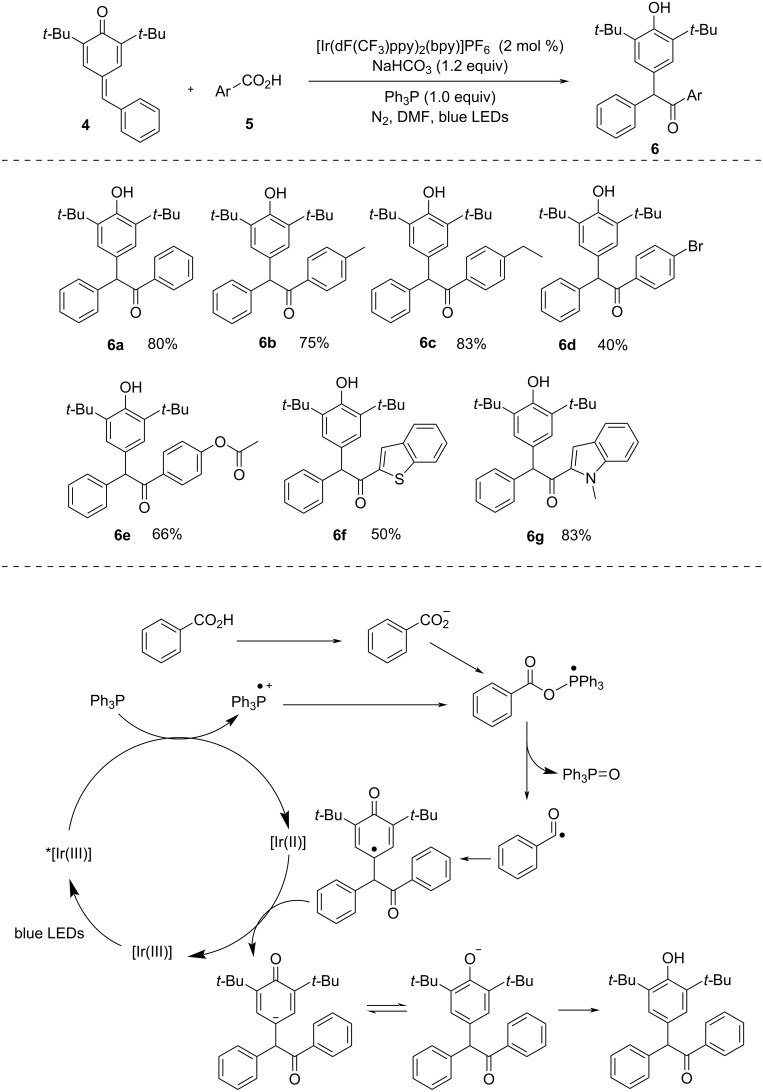
Acylation–aromatization of *p*-quinone methides using carboxylic acids.

α,α′-Diarylated ketones serve as crucial building blocks in the construction of both natural and synthetic compounds with significant biological relevance. In 2022, Yu et al. [29] showcased a very mild procedure for the effective synthesis of α,α′-diarylated ketones (Scheme 2). Compared to previous procedures, this methodology was a significant improvement as it did not require an excessive amount of additives or high temperature. The methodology was applicable to various carboxylic acids with electron-donating as well as electron-withdrawing substituents but unfortunately, aliphatic acids were not effective in this reaction. In addition, several control experiments, such as fluorescence quenching and Stern–Volmer studies, were done to analyze the SET transfer process of PPh_3_ and quenching of photoexcited *[Ir(dF(CF_3_)ppy)_2_(bpy)]PF_6_ by PPh_3_.

**Scheme 3:**
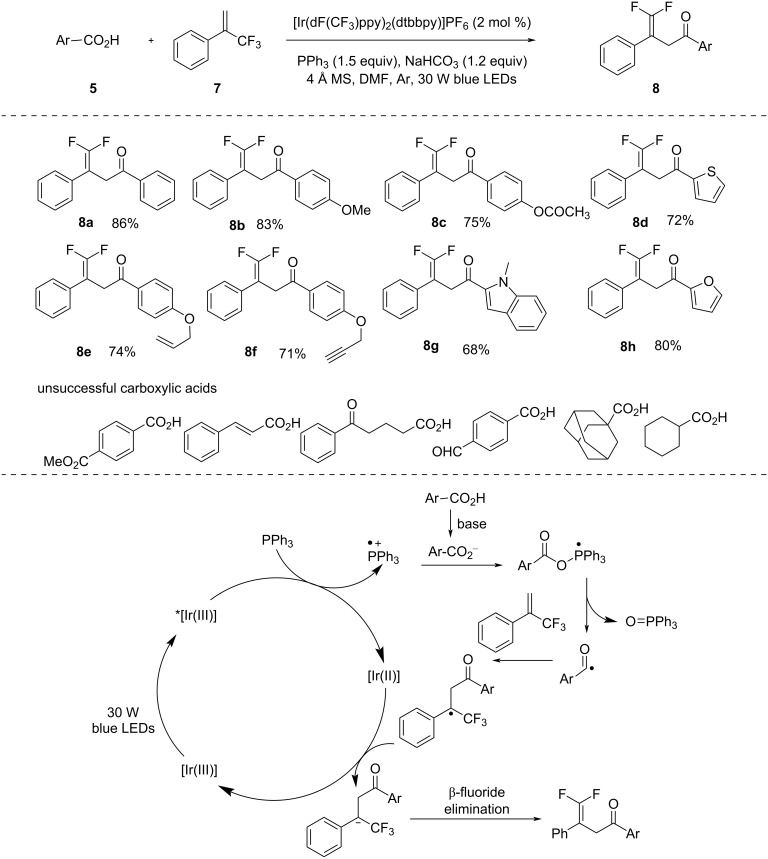
Visible-light-induced deoxygenation–defluorination for the synthesis of γ,γ-difluoroallylic ketones.

Fluorinated organic compounds are widely used in pharmaceuticals and pesticides. Therefore, it is crucial to diversify organic scaffolds by addition of fluorinated groups or by defluorination. In 2020, Wang and co-workers [30] demonstrated the photomediated synthesis of γ,γ-difluoroallylic ketones by reacting trifluoromethyl alkenes and acids in the presence of PPh_3_ additive and iridium photocatalyst in basic medium (Scheme 3). This methodology was suitable for a wide range of carboxylic acids in the presence of alkene, alkyne, halogen, and ether moieties. *N*-Boc-protected amines and esters also provided a good to excellent yield. Unfortunately, α,β-unsaturated carboxylic acids and aliphatic carboxylic acids were ineffective using this method.

**Scheme 4:**
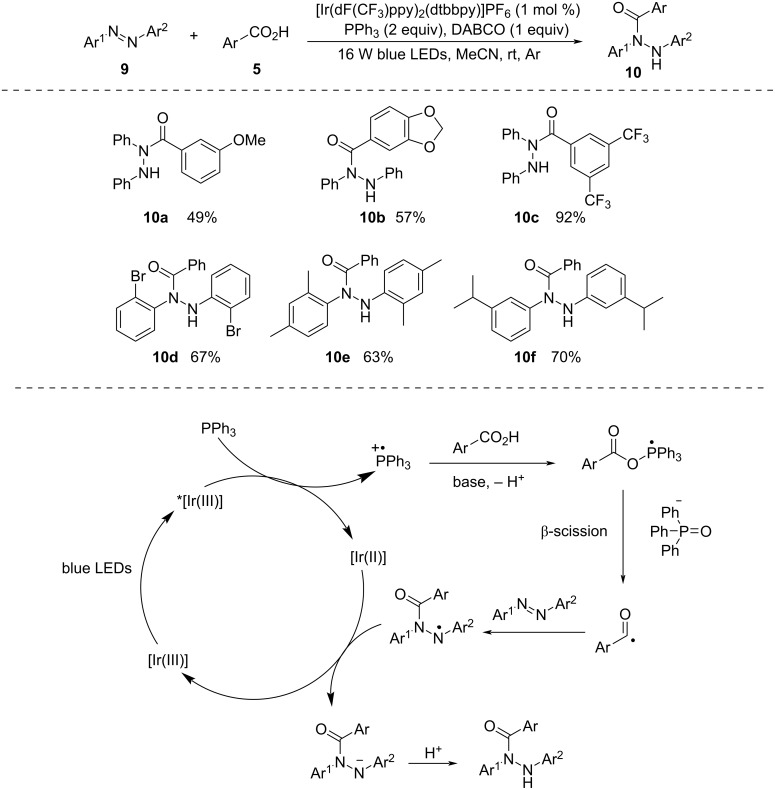
Photochemical hydroacylation of azobenzenes with carboxylic acids.

In 2024, Liu and co-workers [31] introduced a photocatalytic hydroacylation of azobenzenes employing acids as hydroacylating reagents (Scheme 4). The reaction progressed smoothly, involving the cleavage of the C–O bond using a photogenerated phosphoranyl radical. The methodology demonstrated an excellent compatibility with a wide range of azobenzenes and carboxylic acids, yielding diverse *N*,*N*′-diarylhydrazides. This class of compounds is traditionally challenging to synthesize, making this approach a valuable alternative. The method offers a mild and effective way to create *N*,*N*′-diarylhydrazides using easily accessible starting materials.

Initially, photoexcitation of the iridium photocatalyst [Ir(dF(CF_3_)ppy)_2_(dtbbpy)]PF_6_ leads to excited-state *[Ir(III)], *E*_red_ (*[Ir(III)]/[Ir(II)]) = +1.21 V, possessing sufficient energy to oxidize PPh_3_, forming the triphenylphosphine radical cation. Subsequently, benzoic acid undergoes deprotonation facilitated by a base, producing benzoate. This benzoate then reacts with the triphenylphosphine radical cation, resulting in the formation of the phosphoranyl radical intermediate, which undergoes β-scission, leading to the formation of a benzoyl radical, accompanied by the liberation of a triphenylphosphine oxide molecule. After this, the addition of the benzoyl radical to azobenzene results in the generation of a nitrogen-centered radical. This radical is then subjected to reduction by the reduced photocatalyst, producing the nitrogen-centered anion intermediate. Ultimately, the protonation of this anion gives rise to the desired product.

**Scheme 5:**
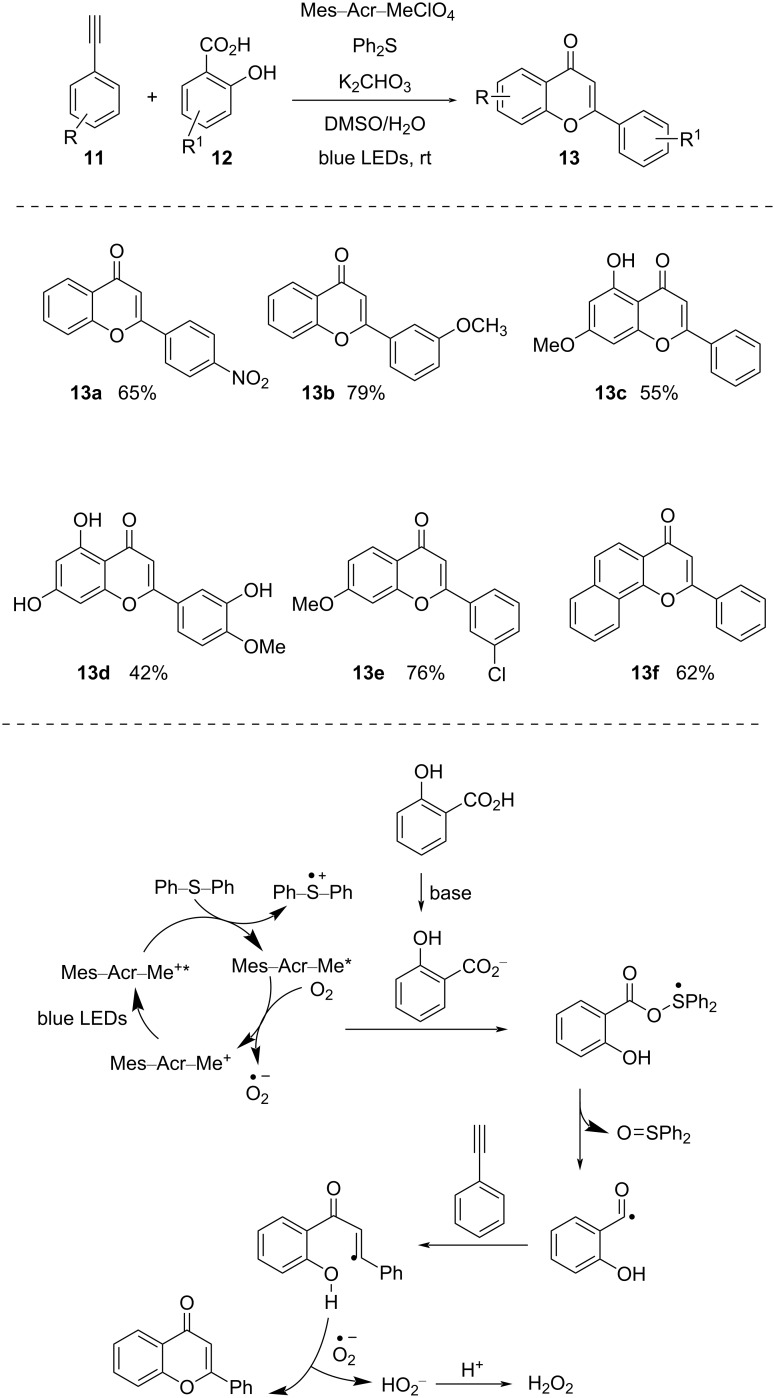
Photoredox-catalyzed synthesis of flavonoids.

The photomediated formation of acyl radicals directly from acids mostly employs DMDC or phosphines (e.g., PPh_3_, PMe_2_Ph) as additives and [Ir(III)] as photocatalyst. In 2022, Chu and co-workers [32] developed a protocol to form acyl radicals directly from acids utilizing Ph_2_S as activator and the organic dye Mes–Acr–MeClO_4_ as photocatalyst (Scheme 5). They demonstrated intermolecular radical cyclization of *o*-hydroxybenzoic acid derivatives with terminal alkynes to afford flavone derivatives. Here, functionally diverse flavonoids were synthesized in moderate to excellent yield by reacting various salicylic acid derivatives and aryl acetylenes.

Due to irradiation with blue light, Mes–Acr–Me^+^ gets excited to Mes–Acr–Me^+^* and takes up a single electron from Ph_2_S. The reaction of the diphenyl sulfide radical cation with carboxylate and successive acyloxy C–O bond cleavage forms diphenyl sulfoxide and an acyl radical. This acyl radical eventually leads to the formation of expected product.

### Reactions involving alkyl radical obtained via C–O bond cleavage

#### C–O bond activation of prefunctionalized alcohols

Alkyl radicals play a crucial role as intermediates in various chemical transformations involving C–H, C–C, and C–heteroatom bond formations. The best known technique for the creation of alkyl radicals is the homolytic cleavage of the C–X bond of alkyl halides by toxic tin hydride [17]. Later, various efforts have been made to replace toxic tin hydrides with other reagents [33–42]. However, these protocols have a limited scope and suffer from prefunctionalization and waste generation. Photons are considered the greenest reagent in organic synthesis. Thus, photomediated alkyl radical generation from easily accessible alcohols for organic synthesis is highly interesting. However, direct C–O bond activation of alcohols by visible light is limited due to the large redox potential and the high C–O bond energy. As such, the conversion of alcohols to redox-active groups is necessary to tackle this issue. In this section, we will discuss various types of prefunctionalized alcohols that are used under visible-light photoredox conditions to generate alkyl radicals by homolysis of C–O bonds.

**Scheme 6:**
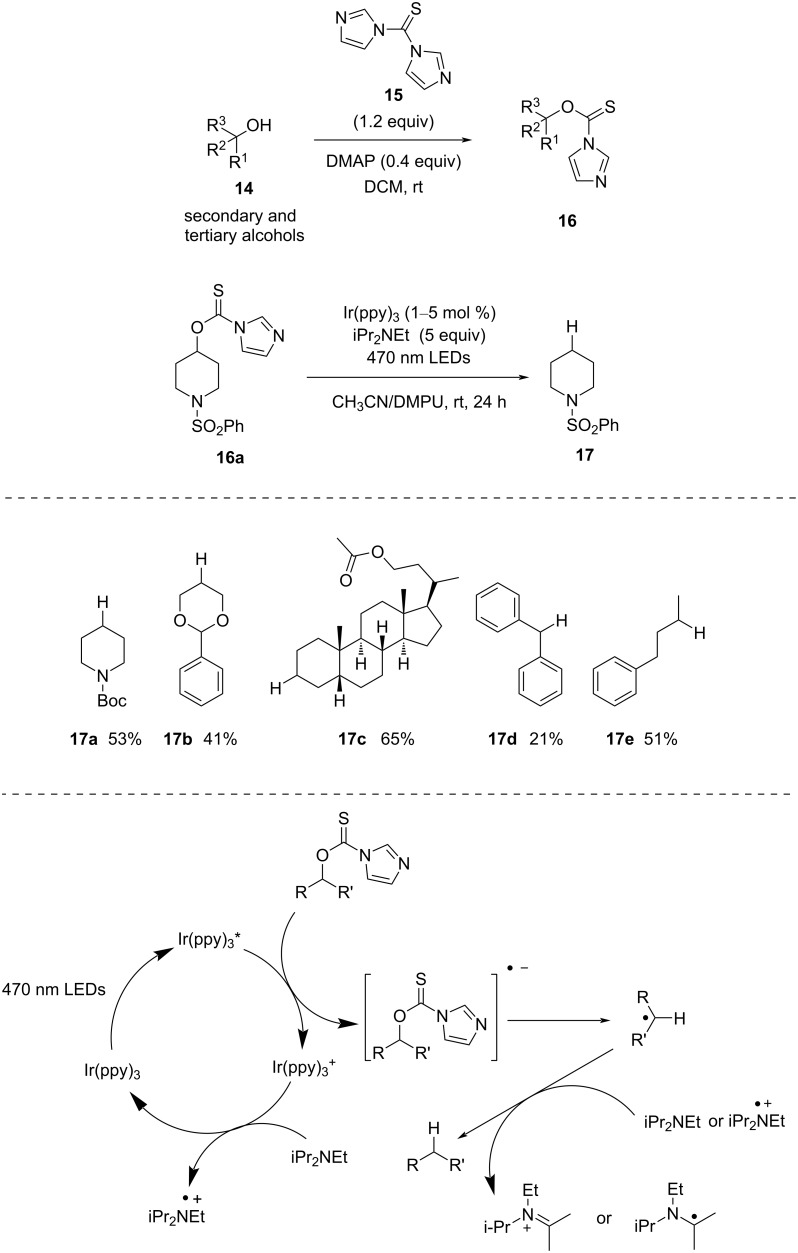
Synthesis of *O*-thiocarbamates and photocatalytic reduction of *O*-thiocarbamates.

**Thiocarbonyl:** In 2014, Ollivier and co-workers [43] demonstrated visible-light-mediated iridium-catalyzed reduction of thiocarbonyl derivatives derived from alcohols. The thiocarbonyl derivatives were prepared by reaction of thiocarbonyldiimidazole (TCDI, **15**) with alcohols in the presence of a catalytic amount of DMAP (0.4 equiv). TCDI is a very popular substrate for such reaction types and was first introduced by Barton and McCombie (Scheme 6) [20].

In this process, the catalyst is first excited and then transfers one electron to the thiocarbamate moiety to form a thiocarbamate radical anion, with change in oxidation state from III to IV. Next, the sacrificial electron donor Hünig base successfully converts [Ir(IV)] to [Ir(III)], with concurrent formation of an amine radical cation. Then, the photogenerated thiocarbamate radical anion forms the alkyl radical by homolytic cleavage of the C–O bond. Next, the H-atom abstraction from iPr2NEt or the from the corresponding radical cation furnishes the desired product.

**Scheme 7:**
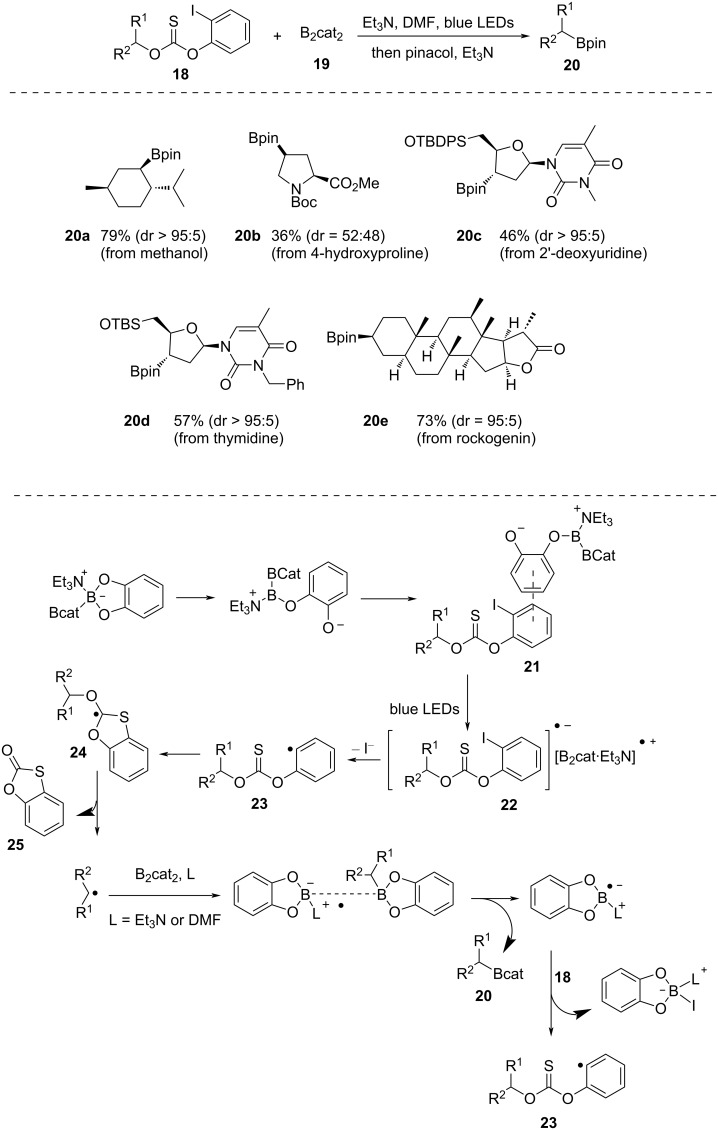
Deoxygenative borylation of alcohols.

In 2019, Aggarwal and co-workers [44] described a method to convert aliphatic alcohols into boronic esters. At first, aliphatic alcohols are functionalized into 2-iodophenyl thionocarbonates that can facilitate a visible-light-mediated Barton–McCombie radical deoxygenation (Scheme 7). The reaction is free from photocatalyst, radical initiators, or conventional metal hydrides, such as tin or silicon hydrides. The reaction mechanism is interesting since first, a Lewis acid–base adduct is generated by interaction of Et_3_N with a boron atom of bis(catecholato)diboron (B_2_cat_2_, **19**). As a result, one of the catecholate ligands experiences an increase in electron density, which facilitates a π–π interactions with the aryl iodide system and ultimately results in the production of an electron donor–acceptor (EDA) complex **21**. Photoexcitation of this EDA complex furnishes an aryl iodide radical anion and a radical cation complex **22**. Then, the elimination of iodide leads to the formation of **23**, which further undergoes 5-*endo*-trig cyclization, followed by fragmentation to produce an alkyl radical and a cyclic thiocarbonate **25**. These alkyl radicals then interact with B_2_cat_2_ (**19**) to produce a variety of structurally intricate boronic esters. The reaction occurs in DMF solvent and requires ca. 18 h to finish upon irradiation with blue LEDs. The catecholboronic esters produced at first are transesterified into pinacol borane by addition of pinacol and triethylamine. The reaction proved to be useful for a wide variety of substrates, such as borneol, menthol, epiandrosterone, hecogenin, rockogenin, and tigogenin. All of them were successfully converted into boronic esters with high diastereoselectivity.

**Scheme 8:**
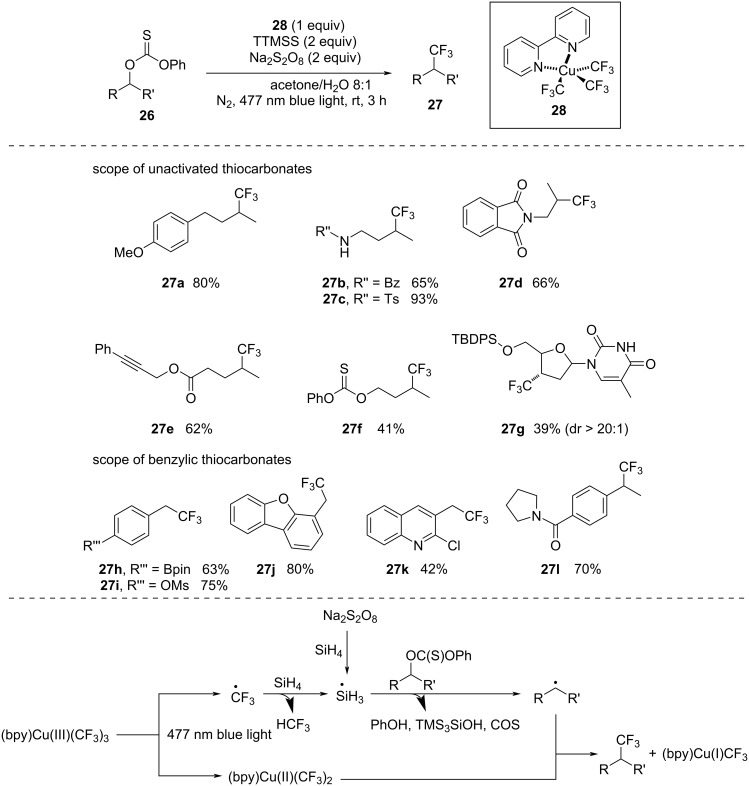
Trifluoromethylation of *O*-alkyl thiocarbonyl substrates.

In 2021, Cook and co-workers [45] unveiled the photomediated trifluoromethylation of alcohols by converting alcohols into thiocarbonates (Scheme 8). This copper-mediated deoxygenative trifluoromethylation technique worked with both benzylic and unactivated thiocarbonates. The proposed mechanism starts with the homolysis of (bpy)Cu(III)(CF_3_)_3_ by blue-light irradiation, which produces CF_3_ radicals and (bpy)Cu(II)(CF_3_)_2_. Subsequently, the interaction between the CF_3_ radical and silane furnishes a Si-based radical, which in turn reacts with the thiocarbonate to form an alkyl radical. Finally, a coupling reaction of the alkyl radical with (bpy)Cu(III)(CF_3_)_3_ leads to the formation of the trifluoromethylation product and a Cu(I) species.

**Scheme 9:**
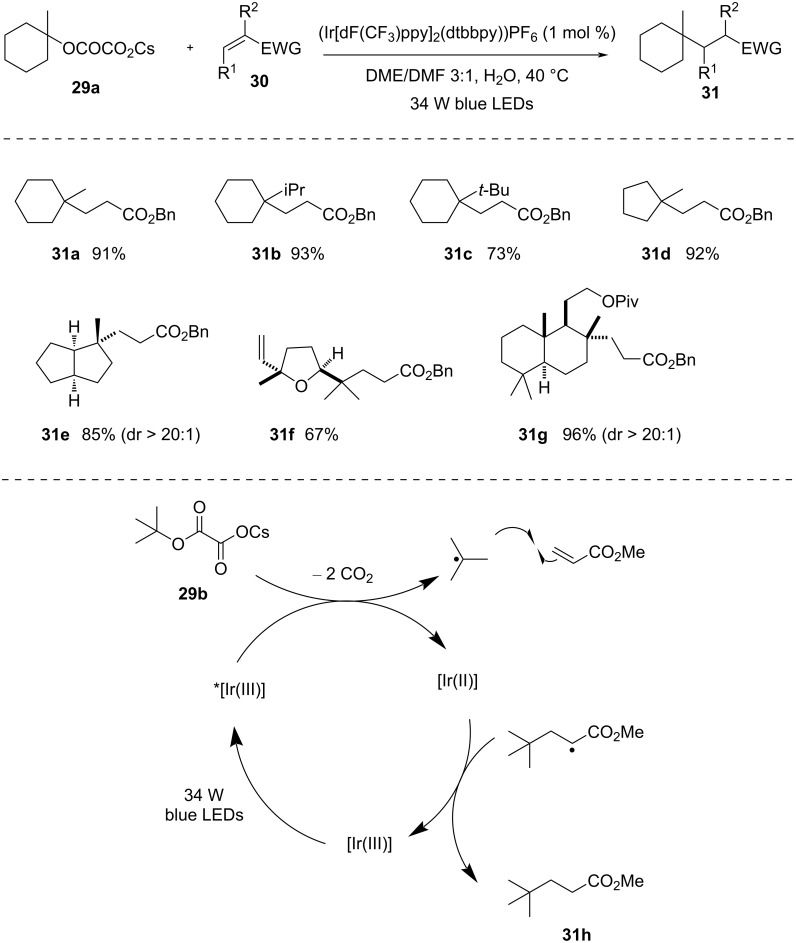
Redox-neutral radical coupling reactions of alkyl oxalates and Michael acceptors.

**Oxalates:** In 2015, Macmillan and co-workers [46] utilized oxalates as activating groups for alcohols. Alkyl oxalates were effectively converted into useful radicals, catalyzed by an iridium complex under visible-light photoredox conditions (Scheme 9). This new approach does not require any sacrificial use of reductants or oxidants and is entirely redox-neutral. The authors have shown that simple cesium alkyl oxalates of tertiary alcohols can easily couple with electron-deficient alkenes in the presence of visible light. Initially, the [Ir(III)] photocatalyst is excited to the long-lived higher-energy state *[Ir(III)]. Then, the excited-state photocatalyst oxidizes the cesium alkyl oxalate via SET, followed by elimination of two carbon dioxide molecules, generating a tertiary alkyl radical that easily combines with an electron-deficient alkene, providing the product. This protocol was well compatible with a wide range of acceptor components, such as various acrylates, α,β-unsaturated acids, enones, enals, acrylamides, vinyl phosphonates, and vinyl sulfones. Various cesium salts of oxalates also performed well using this protocol. Isopropyl and *tert*-butyl groups present in an adjacent position of oxalates do not disturb the reaction and provide the desired products with good yield. Cyclopentanol-derived oxalates, some heterocyclic oxalates, and natural-product-derived oxalates were also compatible with this method.

**Scheme 10:**
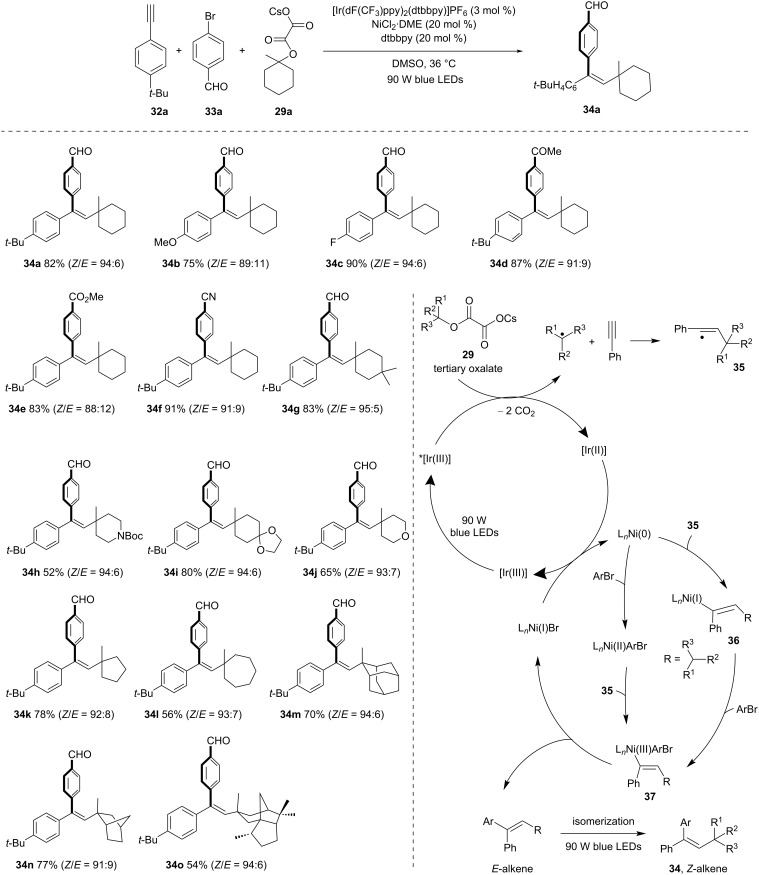
Visible-light-catalyzed and Ni-mediated *syn*-alkylarylation of alkynes.

In 2018, Chu and co-workers [47] devised an elegant protocol for achieving *syn-*alkylarylation of terminal alkynes using tertiary alkyl oxalates and aryl bromides (Scheme 10). This is achieved through the synergistic combination of photoredox and nickel catalysis. This approach facilitates the formation of diverse trisubstituted olefins with outstanding regioselectivity and *syn*-stereoselectivity. The proposed mechanism involves C–O bond activation of tertiary oxalates. It requires [Ir(dF(CF_3_)ppy)_2_(dtbbpy)]PF_6_ and NiCl_2_⋅DME along with dtbbpy ligand. The reaction commences with single-electron oxidation of cesium oxalate initiated by *[Ir(III)] photocatalyst. This transfer leads to the elimination of two CO_2_ molecules and results in a tertiary alkyl radical, which eventually reacts with an alkyne to yield a vinyl radical **35**. Later, the addition of Ni(0) and ligand to the vinyl radical **35** gives the intermediate **36**. This intermediate undergoes oxidative addition with aryl bromide to produce Ni(III) species **37**. A final reductive elimination gives the desired alkene and Ni(I). The two catalytic cycles are finally completed by single-electron reduction of [Ni(I)] by [Ir(II)], which regenerates [Ni(0)] and ground-state [Ir(III)]. Cyclic oxalates readily form the corresponding alkyl radicals under iridium photocatalysis. The generated alkyl radicals then undergo the desired addition with alkyne to produce alkenyl radicals that via Ni-catalysed coupling reactions with aryl bromides form trisubstituted alkenes *Z*-selectively. Internal alkynes are not suitable for this transformation due to the steric reason, but terminal arylalkynes bearing electron-donating and electron-withdrawing substituents were well compatible with this method. The procedure is limited to electron-withdrawing and electron-neutral aryl halides. The presence of a conjugated substituent in the *p*-position of an aryl halide is crucial for achieving good *syn*-stereoselectivity.

**Scheme 11:**
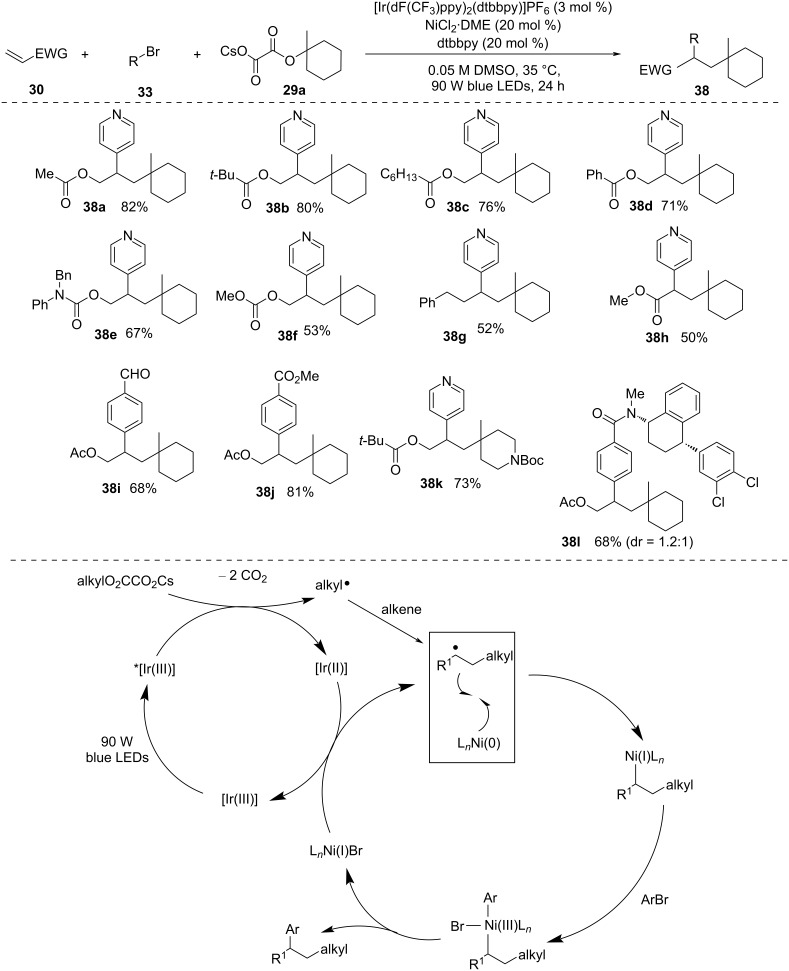
1,2-Alkylarylation of alkenes with aryl halides and alkyl oxalates.

In 2019, using a similar concept, they reported 1,2-alkylarylation of alkenes with alkyl oxalates and aryl bromides under visible-light photoredox and Ni catalysis (Scheme 11) [48]. This protocol was applicable to a wide range of substrates, such as nonactivated alkenes, heteroatom-substituted alkenes, and conjugated alkenes. Using alkyl oxalates obtained from readily available alcohols facilitated the construction of intricate alkyl compounds. However, only tertiary alkyl oxalates were applicable to this methodology.

**Scheme 12:**
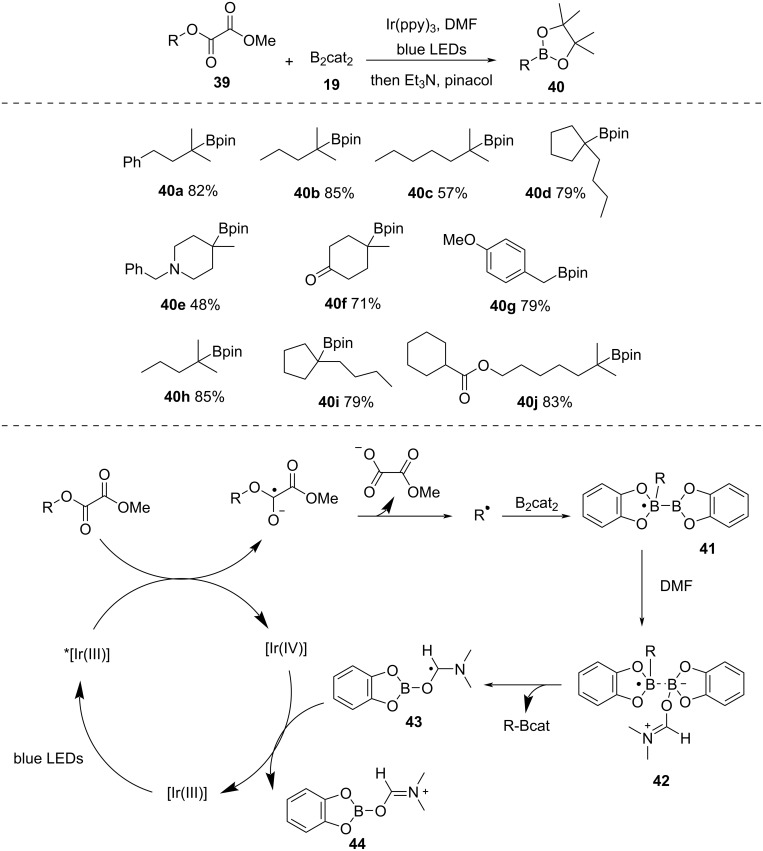
Deoxygenative borylation of oxalates.

In 2019, Studer et al. [49] reported photoinduced C–O borylation of tertiary alcohols using oxalates as a radical source and Ir(ppy)_3_ as a photocatalyst (Scheme 12). At first, the tertiary alcohols were functionalized using Barton pyridine-2-thione-*N*-oxycarbonyl (PTOC) esters for *tert*-alkyl radical generation. Next, the oxalates were utilized for borylation in DMF with the help of photoexcited Ir(ppy)_3_. The suggested mechanism begins with the formation of photoexcited *[Ir(III)], which promotes SET to oxalate and generates an oxalate radical anion and [Ir(IV)]. The fragmentation of the oxalate radical anion produces an alkyl radical. The radical subsequently undergoes addition to B_2_cat_2_ (**19**) to produce the boryl radical **41**. Here, the choice of solvent is also important. The interaction between DMF and the boryl radical **41** assists B–B bond scission to furnish the target borylated products and an intermediate **43**, which is oxidized by [Ir(IV)], regenerating the [Ir(III)] catalyst and completing the catalytic cycle.

**Scheme 13:**
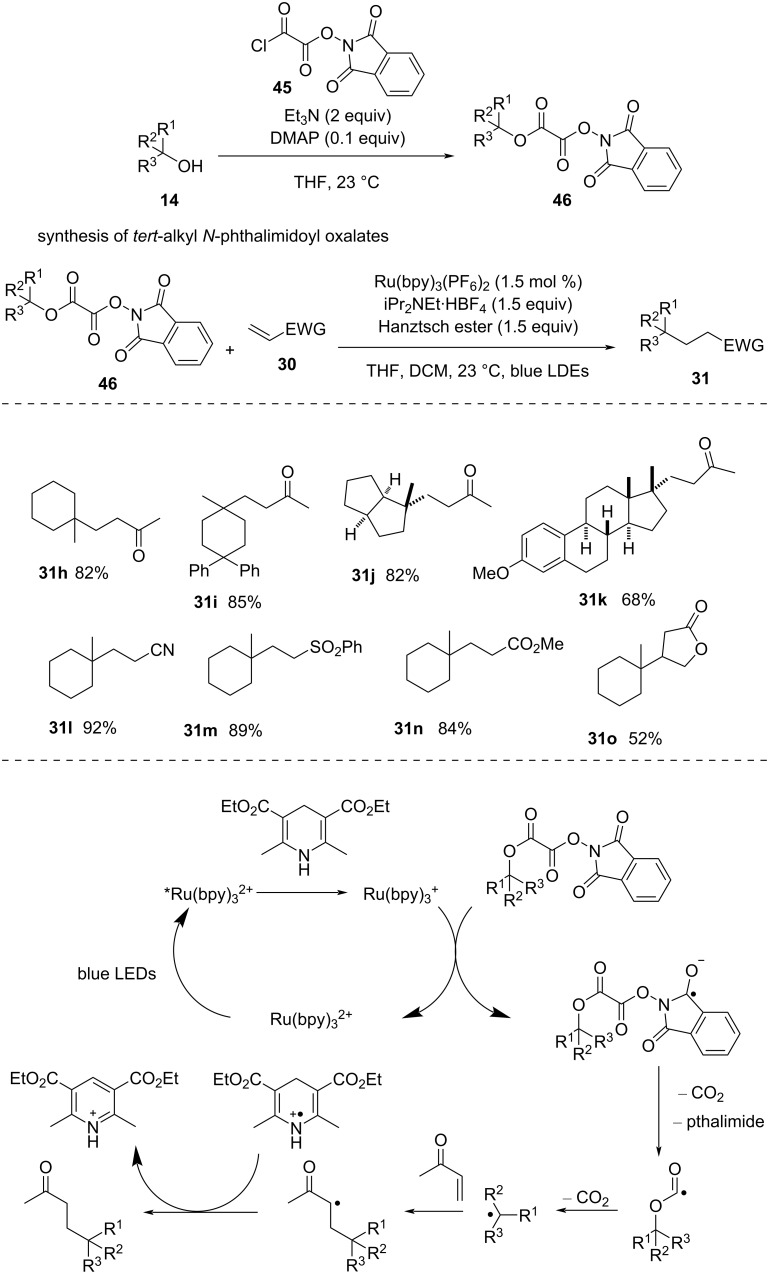
Coupling of *N*-phthalimidoyl oxalates with various acceptors.

Overman and co-workers [50] used *tert*-alkyl *N*-phthalimidoyl oxalates to produce alkyl radicals that were further reacted with various Michael acceptors (Scheme 13). The photocatalyst Ru(bpy)_3_^2+^ and Hantzsch ester were essential for the success of the reaction. The reaction was well compatible with various *N*-phthalimidoyl oxalates (i.e., **31h**–**k**) as well as electron-deficient alkenes (i.e., **31l**–**o**).

**Scheme 14:**
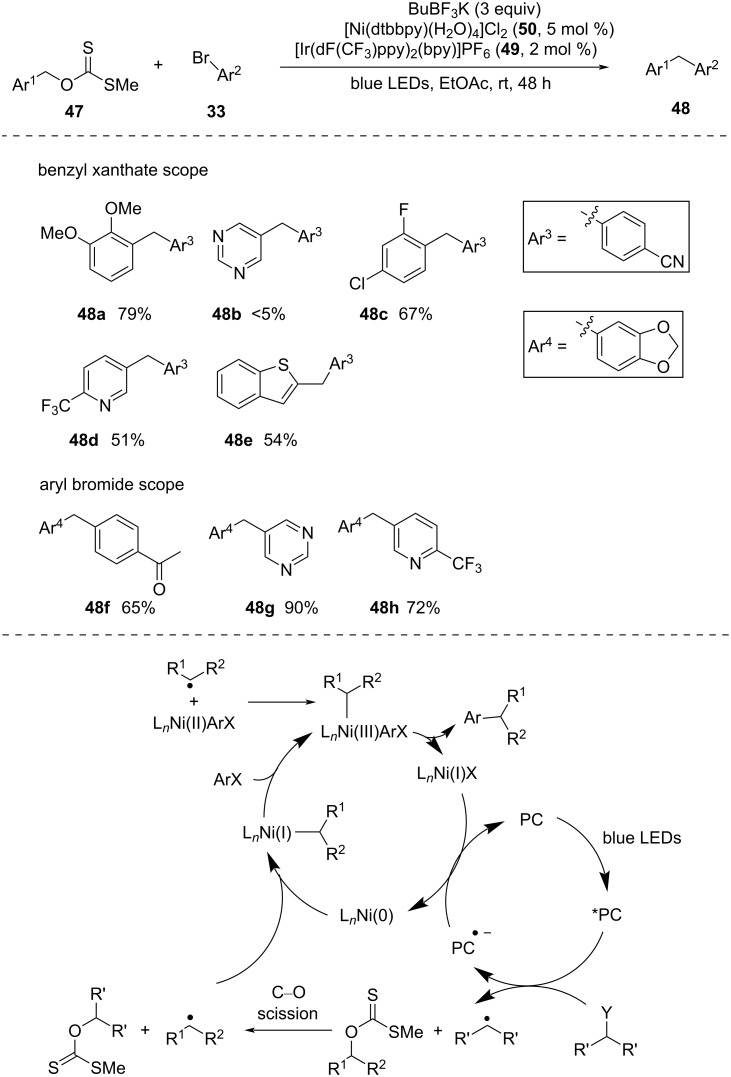
Cross-coupling of *O*-alkyl xanthates with aryl halides via dual photoredox and nickel catalysis.

**Xanthates:** In 2017, Molander and co-workers [51] introduced a C(sp^3^)–C(sp^2^) cross-coupling reaction of benzyl radicals generated from *o*-benzyl xanthate esters with aryl bromides via dual photoredox and nickel catalysis (Scheme 14). *sec*-BuBF_3_K was found to be the best radical precursor for generating the alkyl radicals that initiated the C–O bond cleavage of *O*-benzyl xanthate esters to provide benzyl radicals. Next, the benzyl radicals underwent nickel-catalyzed cross-coupling reactions with aryl halides to deliver the desired cross-coupled products. Interestingly, in absence of any xanthate, *sec*-butyl radicals underwent cross-coupling reactions with aryl halides to form *sec*-butyl arenes, whereas in the presence of xanthate, no undesired *sec*-butyl arenes were generated. This underpinned the formation of *sec*-butyl radicals in the system that rapidly reacted with *O*-benzyl xanthates before participating in the nickel-catalyzed cross-coupling reactions. Precatalyst [Ni(dtbbpy)(H_2_O)_4_]Cl_2_ (**50**) and photosensitizer [Ir(dF(CF_3_)ppy)_2_(bpy)]PF_6_ (**49**) were found to be the best choices for a smooth reaction progress.

**Scheme 15:**
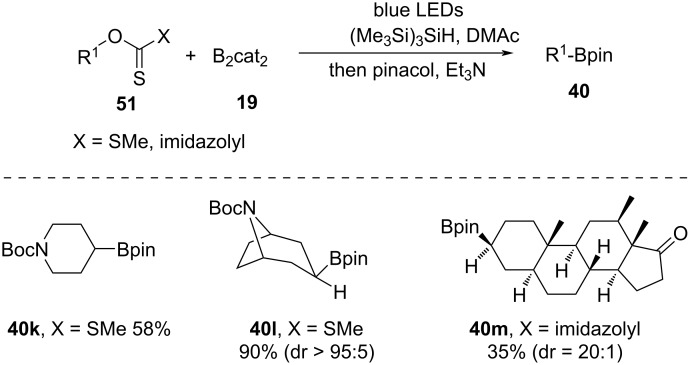
Deoxygenative borylation of secondary alcohol.

In 2019, Studer et al. [49] reported a borylation reaction of alcohols by conversion into xanthates that act as alkyl radical source via photocatalytic deoxygenation (Scheme 15). Therein, silane was used as radical agent for the reduction of xanthates. Under blue-light irradiation, xanthates were reacted with B_2_cat_2_ (**19**) in dimethylacetamide (DMAc), providing the borylated product in decent yield. The methodology was metal-free and did not require conventional heating. Nevertheless, the borylation process was limited to secondary alcohols. To address the instability of the catecholate products, they were converted in situ to the Bpin esters by introducing pinacol and Et_3_N into the reaction mixture.

**Scheme 16:**
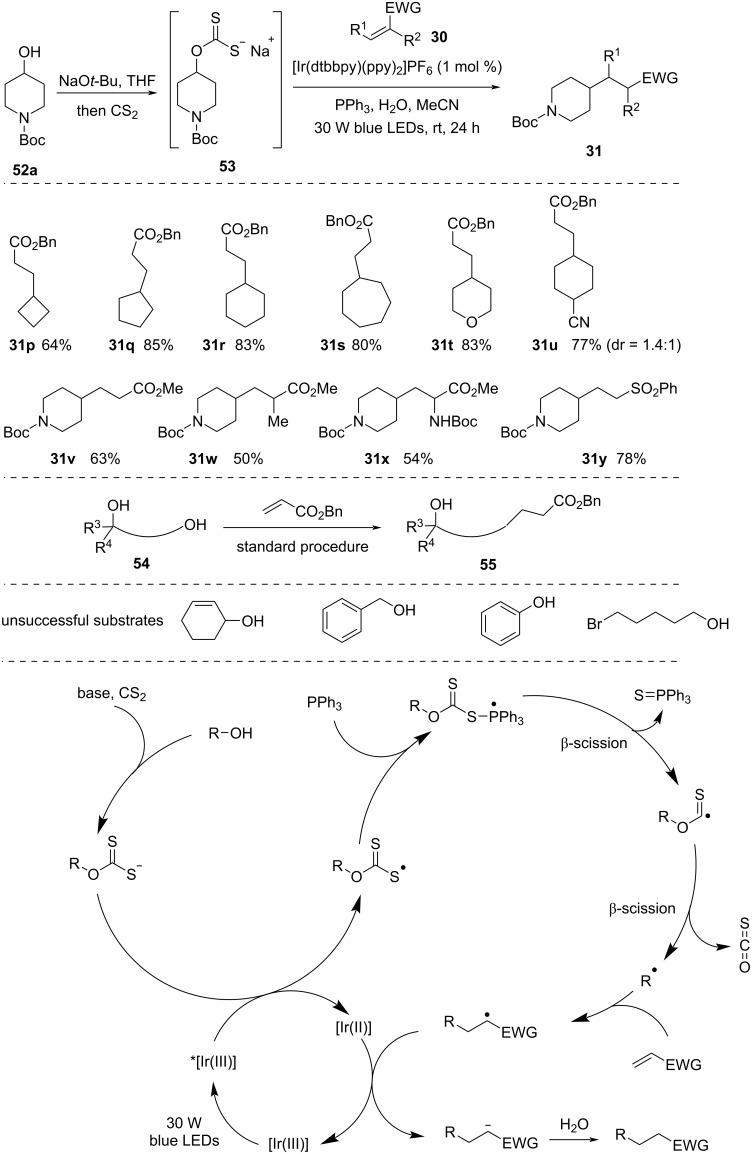
Deoxygenative alkyl radical generation from alcohols under visible-light photoredox conditions.

In 2021, Wu and co-workers [52] developed a method in which alkyl radicals were generated via photocatalytic deoxygenation of alcohols (Scheme 16). This one-pot strategy involved the reaction of xanthates formed in situ with electron-deficient alkenes under visible-light photoredox conditions in the presence of PPh_3_. This approach did not require multistep synthesis of starting materials. In addition, alcohol groups in polyols could be converted rather selectively in the order tertiary alcohol< secondary alcohol < primary alcohol.

They also proposed a mechanism, which is outlined in Scheme 16. The first step involves deprotonation of alcohol in the presence of base and nucleophilic attack of CS_2_ to generate a xanthate salt intermediate. Photocatalyst-induced SET from the xanthate intermediate gives rise to a sulfur-centered radical. The xanthate radical combines with PPh_3_, resulting in a phosphoranyl radical. Later, a sequential β-scission and elimination of carbonyl sulfide generates the alkyl radical. This alkyl radical reacts with electrophilic alkene and forms the target product.

**Scheme 17:**
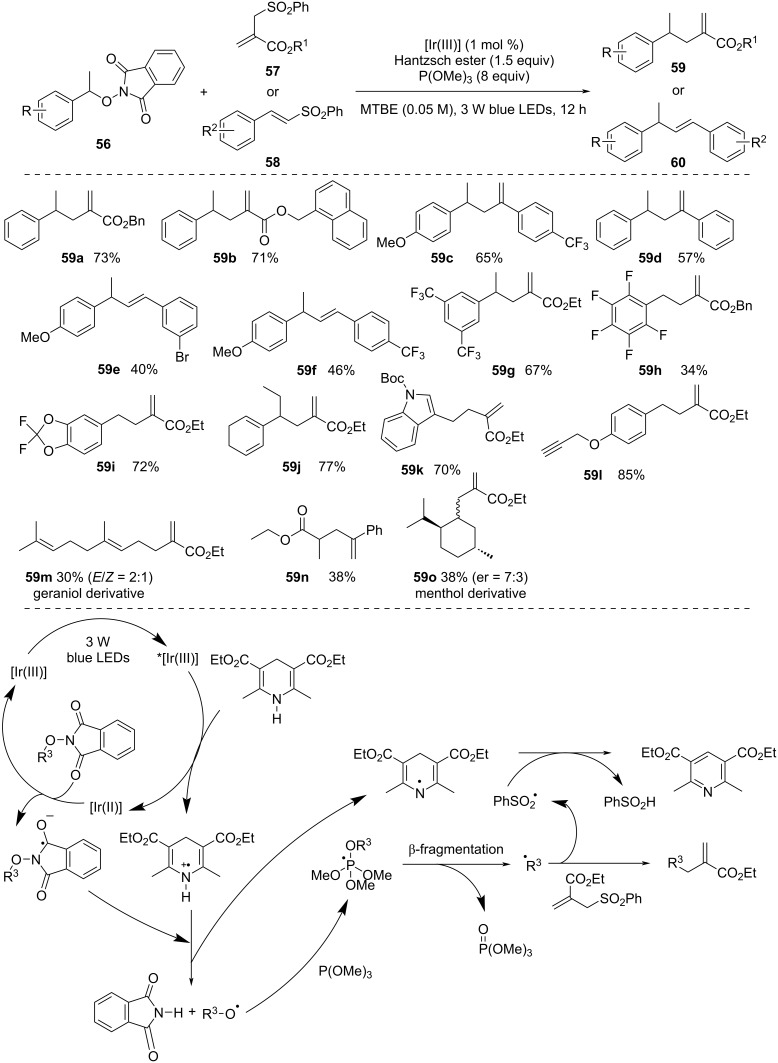
Deoxygenative alkylation via alkoxy radicals against hydrogenation or β-fragmentation.

***N*-Alkoxyphthalimides:** In 2019, Tang and co-workers [53] utilized *N*-alkoxyphthalimides for the generation of alkyl radicals by reacting photogenerated alkoxyl radicals with P(OMe)_3_ (Scheme 17). This strategy provided the alkylation of allyl and alkenyl sulfones with a wide range of *N*-alkoxyphthalimides produced from benzyl alcohols. The protocol also allowed to use *N*-alkoxyphthalimides derived from aliphatic alcohols. However, the reaction was less facile with benzyl alcohols derived *N*-alkoxyphthalimides. The plausible mechanism starts with blue-light excitation of [Ir(III)] to activated *[Ir(III)], which is then reduced by Hantzsch ester to form [Ir(II)]. After SET, the resultant [Ir(II)] species reduces *N*-alkoxyphthalimide to produce the *N*-alkoxyphthalimide radical anion. The Hantzsch ester radical cation further protonates this anion, promoting homolytic N–O bond breaking that yields phthalimide and an alkoxyl radical. This alkoxyl radical can easily extract hydrogen or undergo β-fragmentation. When P(OMe)_3_ is present, the alkoxyl radical combines with it to generate a phosphoranyl radical. This radical easily undergoes β-scission, resulting in the production of an alkyl radical. After that, this alkyl radical reacts with allyl and alkenyl sulfones in an addition–elimination cycle to produce the required product and a benzenesulfonyl radical. The benzenesulfonyl radical abstracts a hydrogen radical from the Hantzsch ester radical to form PhSO_2_H and a pyridine species, thereby completing the catalytic cycle.

#### Direct C–O bond activation of alcohols

Alcohols have a high C–O bond strength and redox potential, making it difficult to directly activate the C–O bonds and produce carbon-centered radicals. Thus, to generate carbon-centered radicals, functionalization of alcohols is required, which requires an additional step. Therefore, it is quite interesting to directly employ alcohols for creating carbon-centered radicals that facilitate photomediated organic transformations. In this section, we will discuss some methods where alcohol C–O bonds were directly activated via photoredox catalysis.

**Scheme 18:**
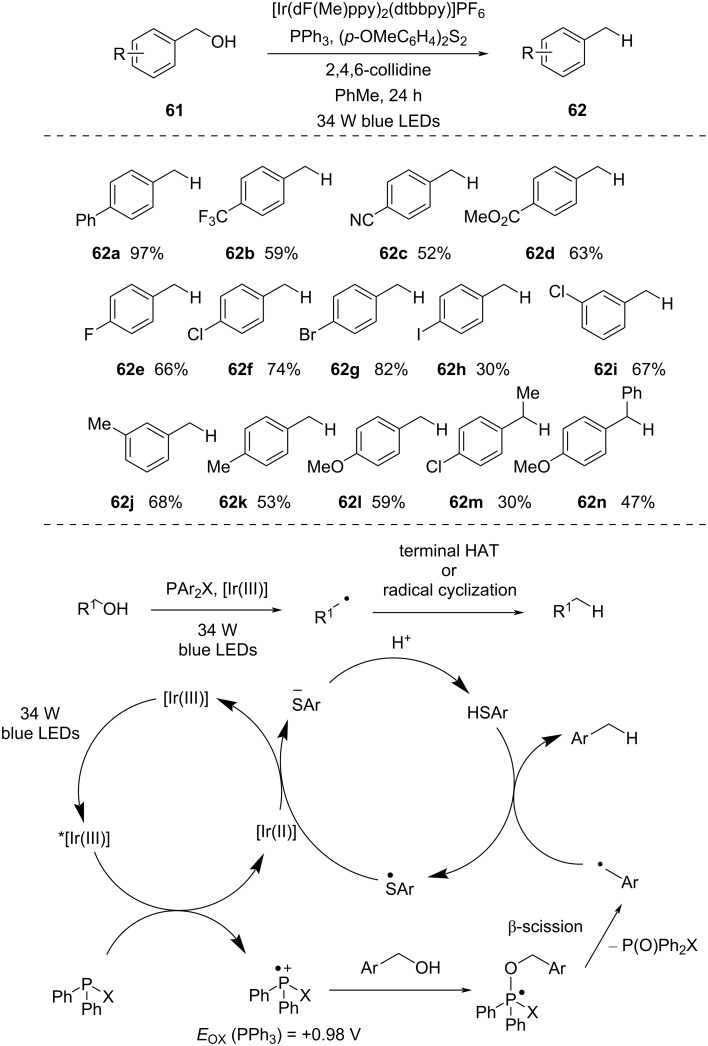
Direct C–O bond activation of benzyl alcohols.

In 2018, Doyle and co-workers [54] documented a catalytic method for the deoxygenation of benzylic alcohols to toluenes, utilizing phosphines and photoredox catalyst under visible-light irradiation (Scheme 18). In this method, they were able to synthesize various hydrocarbons in good yield. Alcohols containing electron-deficient arenes delivered a comparably lower yield (i.e., **62b**–**d**, 52–63%), probably because of the lower nucleophilicity of the alcohols. Halogen atoms present in the *p*-position of alcohols were tolerated well and provided a decent yield (i.e., **62e**–**h**, 30–82%) of the corresponding hydrocarbons. *m*-Substitution also provided a good product yield (i.e., **62i** and **62j**, 67 and 68%). Additionally, electron-rich benzylic alcohols yielded the product in a lower yield (i.e., **62k** and **62l**, 53 and 59%) compared to **62a**. This was due to the generation of electron-rich phosphoranyl radicals, which were more prone to oxidation before undergoing β-scission. The deoxygenation process of secondary benzylic alcohols occurred with decreased efficiency (i.e., **62m** and **62n**, 30 and 47%), in line with the slower addition of a more sterically hindered alcohol to a phosphine radical cation.

The proposed mechanism involves the formation of a phosphine radical cation via SET from photoexcited [Ir(III)] complex. Subsequently, the benzylic alcohol initiates a polar nucleophilic attack on the phosphine radical cation, forming a phosphoranyl radical. This phosphoranyl radical intermediate then undergoes β-cleavage, giving rise to a benzylic radical and triphenylphosphine oxide. A terminal hydrogen atom transfer (HAT), facilitated by an aryl thiol, results in the formation of the desired product with concurrent formation of the thiyl radical. The reduction of the thiyl radical by [Ir(II)] generates a thiolate anion and [Ir(III)]. Finally, the thiolate anion is converted to the aryl thiol via proton transfer to complete the catalytic cycle.

**Scheme 19:**
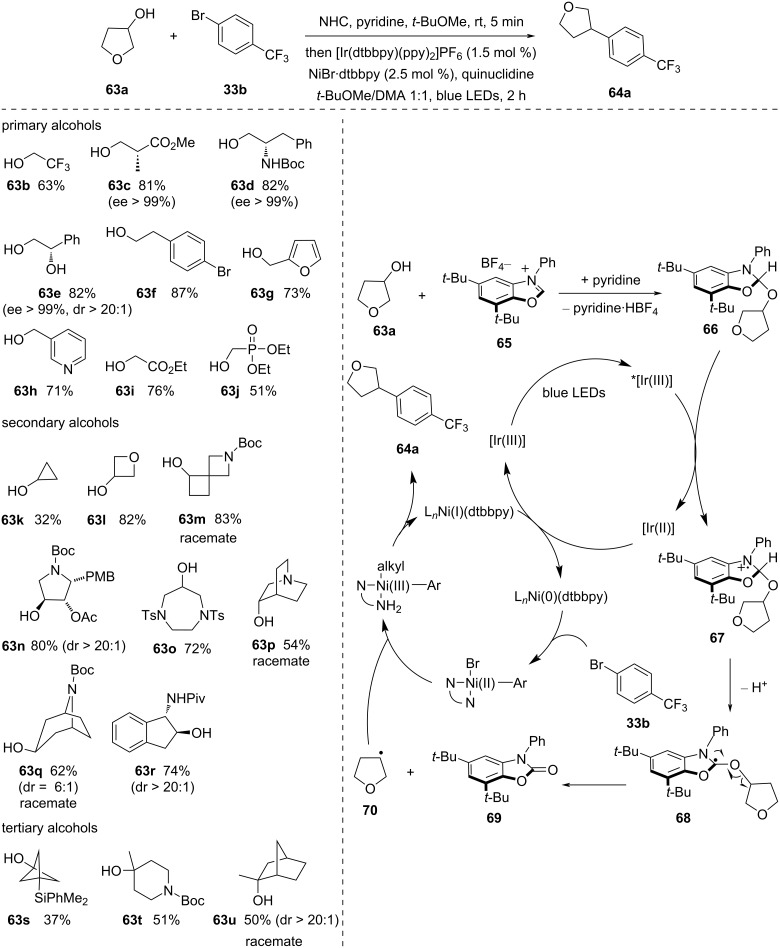
Deoxygenative arylation of alcohols using NHC to activate alcohols.

In 2021, MacMillan and co-workers [55] introduced a cross-coupling reaction of alcohols with aryl halides through metallaphotoredox catalysis (Scheme 19). Therein, alcohols were activated by the use of NHC salts. This activation facilitated the construction of C–C bonds when combined with aryl halide coupling partners. A diverse array of alcohols and various medicinally important aryl and heteroaryl halides reacted well in this protocol.

Under basic conditions, the mechanism involves the condensation of a benzoxazolium salt **80**, creating NHC–alcohol adduct **81**. The [Ir(III)] photocatalyst is excited when exposed to blue light, leading to the formation of a long-lived triplet-excited-state *[Ir(III)] complex.

This excited-state ^*^[Ir(III)] complex can effectively oxidize the nitrogen atom of activated NHC–alcohol adduct **66** via SET. The resulting nitrogen radical cation intermediate **67** weakens the adjacent C–H bond, making it more acidic and susceptible to deprotonation by a suitable base, ultimately yielding an α-amino radical intermediate **68**. This carbon-centered radical, positioned near three heteroatoms, undergoes rapid β-cleavage to produce a carbamate **69** and a deoxygenated alkyl radical **70**. The formation of carbamate byproduct **69**, having a strong C=O double bond, acts as thermodynamic driving factor for the C–O bond homolysis.

**Scheme 20:**
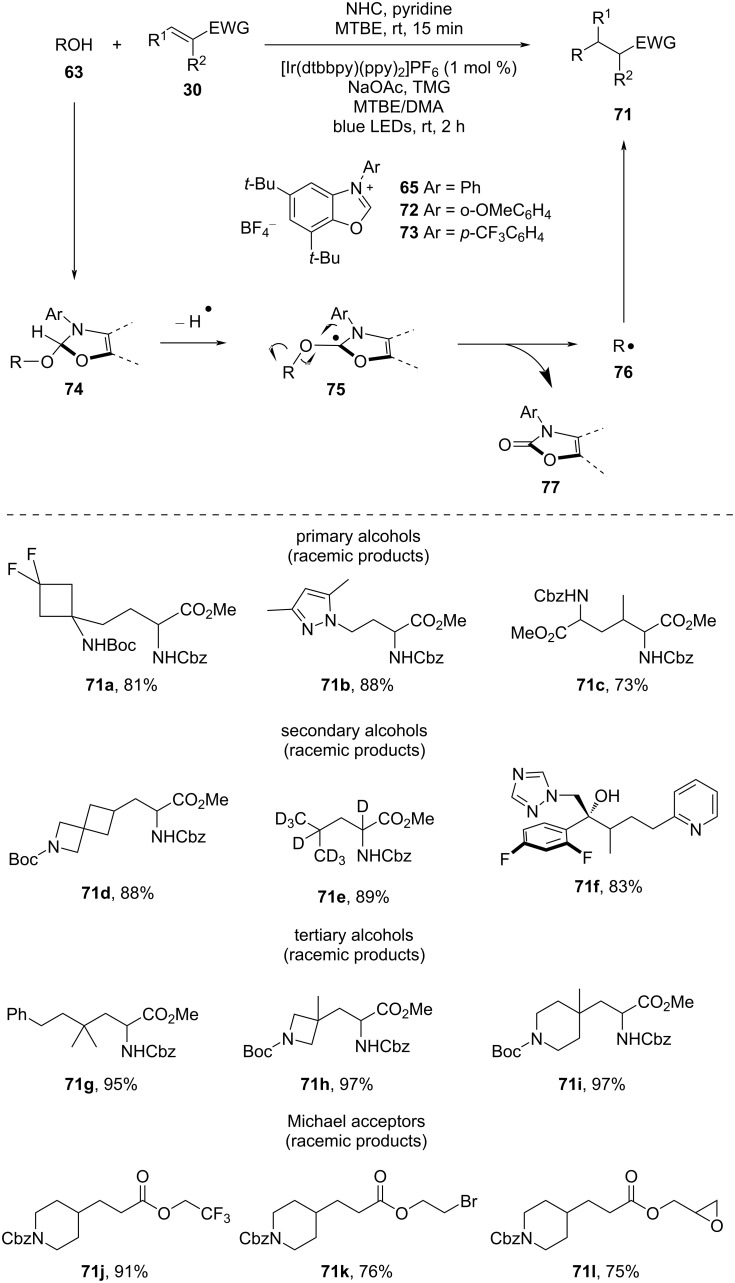
Deoxygenative conjugate addition of alcohol using NHC as alcohol activator.

In 2022, MacMillan and co-workers [56] outlined a comprehensive and direct deoxygenative hydroalkylation method for various types of electrophilic olefins (Scheme 20). This method involved primary, secondary, and tertiary alcohols and was facilitated by the use of a newly developed NHC-based activator. The deoxyalkylation was performed by initially stirring the alcohol substrate with NHC and pyridine in MTBE for 15 min. After that, Michael acceptor, sodium acetate, 1,1,3,3-tetramethylguanidine, and [Ir(III)] photocatalyst in MTBE/DMA were added to the mixture. Then, blue LEDs were used to irradiate the reaction mixture to produce the desired product. The developed protocol was highly attractive as it eliminated waste generation, which was essential for practical use, particularly in late-stage functionalization. For the deoxygenation of primary substrates, which underwent slower β-scission, NHC **72** was found to be most effective. Unstrained secondary alcohols were efficiently activated with the help of NHC **65**. Deoxygenation of sterically congested alcohols, which has been a longstanding challenge in organic synthesis, was achieved by using a more electrophilic alcohol activator NHC **73**. The mechanism was similar to the previous one. Therein, firstly, the alcohol is condensed with the benzoxazolium salt and generated the adduct **74** in situ. Then, the excited photocatalyst is reductively quenched by adduct **74**, followed by proton elimination, generating α-amino radical intermediate **75**. This subsequently undergoes β-cleavage to produce an alkyl radical **76** and an aromatized byproduct. This alkyl radical is then added to electron-deficient alkenes via Giese addition and is followed by reduction, providing the desired product.

#### Other related C–O bond cleavages

**Scheme 21:**
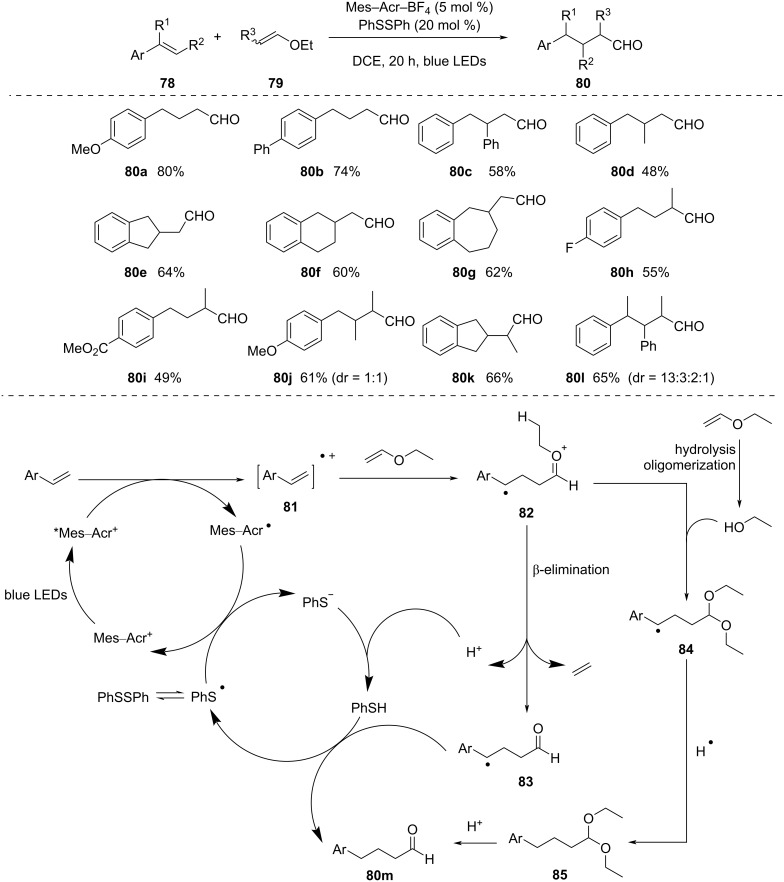
Synthesis of polysubstituted aldehydes.

Recently, the photochemical C–O bond cleavage of ethers in organic transformations has attracted considerable interest. In this context, in 2018, Nicewicz and co-workers [57–58] reported a visible-light-photoredox-catalyzed single-step synthesis of polysubstituted aldehydes using easily accessible olefin substrates (Scheme 21). Styrenes selectively reacted with vinyl ethers in the presence of an acridinium photocatalyst and a diphenyl disulfide HAT catalyst to produce the aldehyde product when exposed to blue LED light. Differently substituted styrenes were examined using this protocol, which produced the aldehyde products in good yield (i.e., **80a**–**d**, 48–80%). Cyclic olefins also performed well under these conditions and generated products with a β-ring moiety (i.e., **80e**–**g**, 60–64%), which would have been challenging to synthesize otherwise. 2-Substituted ethyl vinyl ethers also provided α-branched aldehyde products in decent yield (i.e., **80h**–**k**, 49–66%). An α,β,γ-trisubstituted aldehyde (i.e., **80l**, 65%) was synthesized using an α,β-disubstituted styrene, which could not be produced using the conventional method. The excited photocatalyst *Mes–Acr^+^ oxidized the styrene to produce the extremely electrophilic radical cation **81** and Mes–Acr because of the favorable π–π stacking. Ethyl vinyl ether, which is the most nucleophilic molecule in the reaction, combined with radical cation **81** to form the oxonium radical **82**, which could proceed in two directions: 1) β-elimination, yielding radical **83** and 2) photoinduced breakdown of ethyl vinyl ether and trapping of ethanol, yielding radical **84**. To regenerate the photocatalyst, PhSSPh functioned as an oxidant. After protonation upon the β-elimination step, PhS^−^ contributed a hydrogen atom to both **83** and **84**, alongside regeneration of the HAT catalyst. Lastly, the acetal side product **85** was transformed into the aldehyde by acidic workup.

## Conclusion

The techniques, catalysts, and mechanistic analyses of homogeneous catalytic deoxygenation processes for high-value products have advanced significantly over the last few years. This review presents an extensive overview of recent photocatalysis methodologies in the field of C–O bond activation. As conventional techniques, such as Barton decarboxylation, create hazardous wastes, the use of alternative feedstocks, including alcohols and acids, has been encouraged to achieve sustainability. The recent advancements not only avoid the use of various halide-based reactants but also opened up C–O bond activations in terms of alcohol and acid functionalizations, choice of reactants, etc.

Consequently, in this review, we focused on the advancements in photocatalytic alkyl and acyl radial generation from alcohols and acids. It is highly expected that many groups will explore many new useful synthetic transformations based on renewable feedstocks, such as alcohols and acids, which will likely lead to even more exciting opportunities in the near future.

## Data Availability

Data sharing is not applicable as no new data was generated or analyzed in this study.
